# Factors Associated with an Unhealthy Lifestyle among Adults in Riyadh City, Saudi Arabia

**DOI:** 10.3390/healthcare9020221

**Published:** 2021-02-17

**Authors:** AlJohara M. AlQuaiz, Ambreen Kazi, Turky H. Almigbal, Ali M. AlHazmi, Riaz Qureshi, Khaled M. AlHabeeb

**Affiliations:** 1 Princess Nora Bent Abdullah Research Chair for Women’s Health Research, King Saud University, Riyadh 11321, Saudi Arabia; jalquaiz@ksu.edu.sa (A.M.A.); riaz.qureshi9@gmail.com (R.Q.); kmh-100@hotmail.com (K.M.A.); 2 Department of Family & Community Medicine, College of Medicine, King Saud Medical City, King Saud University, Riyadh 11321, Saudi Arabia; Almogbal@yahoo.com (T.H.A.); aalhazmii@ksu.edu.sa (A.M.A.)

**Keywords:** age, unhealthy lifestyle, physical activity, healthy diet, Saudi Arabia

## Abstract

Background: Unhealthy lifestyles are a global concern. This study measured the prevalence and factors associated with an unhealthy lifestyle in Riyadh city, Saudi Arabia. Methods: An interview-based, cross-sectional study was conducted with 968 males and 2029 females, aged 30–75 years, covering 18 primary health care centers in Riyadh. Multivariate logistic regression analyses were conducted to identify the significant determinants associated with an unhealthy lifestyle. Results: Overall, men were 1.49 (1.28, 1.74) times at higher risk of an unhealthy lifestyle compared to women. Men reporting unhealthy lifestyle were 2.1 (1.3, 3.4) and 1.5 (1.0, 2.6) times more likely than men with healthy lifestyle to cite not enjoying physical activity, lack of social support, and not having enough information about a healthy diet [1.5 (1.0, 2.0)], whereas those ≥ 45 years age group were 30 times less likely to report unhealthy lifestyle [0.7 (0.5, 0.9)]. In contrast, in women aged ≥ 45 years [1.3 (1.1, 1.7)], lack of motivation [1.3 (1.1, 1.7)], feeling conscious while exercising [2.0 (1.4, 2.9)], not enjoying healthy food [1.6 (1.3, 2.1)], and no family support to prepare healthy food [1.4 (1.1, 1.8)] were significantly associated with an unhealthy lifestyle. Conclusions: In a Saudi sample, younger men and older women are at higher risk of an unhealthy lifestyle. In addition to self-motivation, combined strategies to promote physical activity and healthy eating are required to improve lifestyle.

## 1. Introduction

According to the World Health Organization (WHO), the behavioral factors associated with an unhealthy lifestyle include the consumption of a diet with inadequate fruit and vegetables, tobacco smoking, physical inactivity, a sedentary lifestyle, and alcohol consumption [1]. The literature identifies the fact that people who exercise and eat healthy food have a higher chance of living a healthy lifestyle and are at less risk of getting chronic diseases like type 2 diabetes, hypertension, and cardiovascular diseases [2,3].

Globally, the unhealthy lifestyle is becoming more prevalent, with physical inactivity ranging from 43% in the United States of America and the Middle East to 17% in Southeast Asia [3,4]. A national-level study from Saudi Arabia found that the prevalence of total physical inactivity in men versus women, aged 15 to 64 years, was 60% and 73%, respectively [5]. Attractive advertisements and conveniently available fast and processed foods have reduced the consumption of a healthy and fresh diet such as vegetables and fruits [6]. Moreover, the prevalence of smoking has increased, especially among young adults. In addition to cigarettes, the use of other dangerous forms of tobacco smoking, such as water pipe, vapers, e-cigarette, etc., is also increasing [1]. 

An unhealthy lifestyle comprised of sedentary habits is associated with noncommunicable diseases including obesity, cardiovascular diseases, musculoskeletal disorders, depression, and cancers [7]. Previous studies have identified that a lack of resources, increasing age, female gender, family commitments, and adverse socioeconomic conditions are some of the factors associated with physical inactivity and lifestyle in the Arab world [8,9,10]. 

Saudi Arabia is undergoing major developmental reforms, with an emphasis on reducing gender disparity and promoting a healthy lifestyle among the masses [11]. However, a high percentage of the Saudi population follows a sedentary lifestyle, and this is predicted to increase in the future [5,10]. Studies from Saudi Arabia on lifestyle have mainly focused on physical inactivity or obesity, without including eating habits or smoking [5,10,12]. Additionally, these have been hospital-based or institution-based studies conducted on specific populations, hence limiting the generalizability [5,8,9]. Therefore, it is imperative to measure the factors associated with an unhealthy lifestyle in a holistic manner, with representation from all ages and social classes. This study aimed to measure the prevalence and factors of an unhealthy lifestyle among the Saudi population aged 30 to 75 years in Riyadh, Saudi Arabia.

## 2. Materials and Methods

### 2.1. Study Design and Setting

This study was part of a large cross-sectional survey conducted in the capital city of Riyadh, Saudi Arabia, during 2015–2016. This survey aimed to measure the prevalence and correlates of chronic diseases among Saudi men and women aged 30 to 75 years [13]. In order to have representation from all five administrative regions of Riyadh city, 25 primary health care centers (PHCCs) were randomly selected from a list of 105 PHCCs by using a random number generator (RNG—RandomNumberGenerator.com). However, because of logistical issues we were able to include 18 PHCCs in the final analysis.

### 2.2. Study Participants 

Different approaches were adapted to invite the general public for participation in the study. It was extensively advertised in the vicinity of the selected PHCC by placing posters in different places and distributing invitation letters at the selected PHCCs, large shopping malls nearby, local grocery stores, schools, and mosques. The inclusion criteria sought Saudi adults, aged 30 to 75 years, who were permanent residents of Riyadh, whereas non Saudis and pregnant women were excluded from the study. In total, 2997 (968 men and 2029 women) participants fulfilled the eligibility criteria, provided informed signed consent, and completed the interview. 

### 2.3. Data Collection Procedure and Measures

Questionnaire-based interviews were conducted with each participant by a team of Arabic-speaking phlebotomists. The researchers trained the data collectors on conducting interviews, taking anthropometric measurements, and collecting blood samples. A detailed questionnaire was developed, with different sections focusing on physical, mental, and social health. The questionnaire for this study was comprised of sociodemographic characteristics (age, gender, marital status, occupation, income, housing type) and self-reported information on current and past smoking status (number of cigarettes, duration), physical activity, dietary history, and history of physician-diagnosed chronic diseases (including diabetes mellitus, hypertension, dyslipidemia, and arthritis). 

### 2.4. Physical Activity Questionnaire

Physical activity (PA) was measured by the translated validated International Physical Activity Questionnaire (IPAQ-short) [14]. The items in the IPAQ-short are structured to calculate the metabolic equivalent of task (MET) in minutes per week. The MET minutes per week were calculated by multiplying the duration of activity in minutes by the number of days (activity done in a week) and then by multiplying with the pre-assigned values (walking = 3.3 METs, moderate physical activity (PA) = 4.0 METs, and vigorous PA = 8.0). The scores were added to calculate the total MET-minutes per week. 

### 2.5. Dietary Questionnaire

The dietary part comprised questions about commonly consumed food items, including the quantity and frequency of intake in days/weeks/months/year. The quantity was inquired in grams by showing the quarter portion sizes of a standard serving plate. One portion was taken as equivalent to 80 gms and an adult healthy diet should comprise of at least five portions (>400 mg) of fruits and vegetables per day [1,15]. The information related to the remaining food items was utilized elsewhere. 

### 2.6. Definition of Healthy Lifestyle

We have adapted the definition based on the WHO criteria for behavioral risk factors for cardiovascular diseases [1]. The outcome variable “Unhealthy lifestyle” based on behavioral risk factors, was defined as a person reporting <1200 MET-minutes per week, and/or eating <5 portions of vegetables and fruits per day (< 400 gms) and/or being a regular smoker. Participants fulfilling two or all of the above criteria were labeled as having an “unhealthy lifestyle” [1,16,17]. 

### 2.7. Barriers to Physical Activity and Healthy Eating

A list of barriers to physical activity and healthy eating was compiled by retrieving questions from the previous literature and having a discussion with experts [18,19,20]. A validity assessment of all the variables was conducted by two independent researchers. The Arabic translated version of the questionnaire was pretested in the local community. Some of the items that were ambiguous or irrelevant were removed or rephrased according to the sociocultural setting. The final questions numbered 24 and were reviewed by two independent researchers. The barriers belonged to three broad categories, namely, behavioral, sociocultural and environmental. The question was phrased as follows: “Is a specific factor a barrier to you doing physical activity or opting for healthy eating?” The responses were measured as categorical, “yes” (1) vs. “no” (0). Barriers such as no skills to prepare healthy diet were inquired from women only, whereas barrier due to children was inquired from married women only. The Cronbach’s alpha for the physical activity items was 0.72 and for healthy eating items it was 0.80. 

### 2.8. Anthropometric Measurements

A standard protocol was followed to measure anthropometric indices, which included weight measured using an electronic scale (Secca 220, Hamburg, Germany), and height measured using a stadiometer scale. The body mass index (BMI) was calculated using the formula weight (kg) per height (m^2^) [21]. 

### 2.9. Ethical Considerations

The study protocol was reviewed and approved by the Institutional Review Board, King Saud University (E-12-658) and the Institutional Review Board of the Ministry of Health, Dammam (IRB ID MOH0151), Saudi Arabia.

## 3. Data Analysis

The data were analyzed using Statistical Package for Social Sciences (SPSS)® version 21), Armonk, NY. IBM Corp. USA. A descriptive analysis was conducted to measure mean values and the standard deviations for the continuous variables and frequency percentages for the categorical variables. The Pearson correlation coefficient was calculated to measure the association between continuous variables. Chi-square and *p*-values were calculated for categorical variables and a *t*-test was utilized to measure the mean difference between continuous variables. Participants reporting an unhealthy lifestyle were coded as 1, vs. those reporting a healthy lifestyle were coded as 0. Age was divided into categorical variables of 30–44 years, 45–59 years, and 60–75 years. First, univariate analyses were performed to identify the gender differences in factors associated with an unhealthy lifestyle. Similarly, the occupation responses comprised of various professions ranging from doctor, engineer, military services, teacher, allied health services staff, security service, therefore the data was merged to make five major categories, namely, professional, clerical, unemployed, retired and homemaker. The barriers to healthy lifestyle include social and environmental barriers, therefore the male and female analysis was done separately. 

Univariate analysis (without adjusting for potential confounders) was conducted separately for men and women. A unit change in the independent variable causing > 10% change in the estimates (dependent variable) is considered as significant. Hence, variables that were significant in a univariate analysis, suggesting biological plausibility (age, chronic diseases, obesity or causing a ≥10% difference in the estimates, were retained in the final model. Then multivariate binary logistic regression models based on the ENTER method were developed to identify statistically significant variables by generating adjusted odds ratios (aOR) and the 95% confidence interval (CI). All plausible interactions were confirmed before the development of the final model. The Hosmer-Lemeshow test was utilized to predict the goodness of the model fit. The level of significance was set to *p* < 0.05. 

## 4. Results

Table 1 shows the difference between men and women in terms of physical activity, healthy diet intake, and smoking prevalence. The median MET minutes per week (and interquartile ranges) for men and women were 1451 (297, 4158) and 1276 (99, 4350), respectively. A higher percentage of women reported PA < 1200 MET (49.8%) in comparison to men (44.8%) with unadjusted odds ratio (UOR), showing women were 1.22-fold (1.05, 1.42) less likely to be active than men. The dietary habits indicated that 28% (*n* = 272) of men and 25.5% (*n* = 518) (*p* = 0.53) of women were eating <5 portions of vegetables and/or fruit per day. Around one-third of men (28%) were smokers, whereas women, with just 2% reporting as smokers, were at lower risk of smoking (UOR 0.03 (0.02, 0.04) *p* < 0.001). Cigarette was the most common form of smoking reported by men (76%), whereas sheesha smoking was equally reported by both, men and women. Around 20% (*n* = 7) of women had started smoking recently (in the last 1‒2 years), whereas men were found to be chronic smokers (in some cases >50 years). Around 76% (33 out of 44) of women reported smoking <10 cigarettes per day, whereas the majority (41%) of men smoked 10–20 cigarettes/day. Overall, an unhealthy lifestyle was more frequently reported by men as compared to women (56% vs. 44%) (UOR 1.49 (1.28, 1.74), *p* < 0.001).

Table 2 shows the univariate analysis with unadjusted odds ratio and 95% CI for factors significantly associated with an unhealthy lifestyle in men and women. The mean age (±SD) of men and women in this study was 43.1 (±11.7) and 43.8 (±10.9) years, respectively. The majority of participants were married, with men mostly working as doctors, engineers, or in the military and women as teachers, secretaries, or allied health staff. The average number of children reported by married women was five (±3.0). About 27% (*n* = 542) of women were postmenopausal. Age distribution found that more men aged 30-44 years (63% vs 55%) reported unhealthy lifestyle, whereas, more women aged 60-75 years (14% vs 8.6%) reported an unhealthy lifestyle. The mean BMI was not significantly different between the healthy and unhealthy groups for both men and women (men 29.7 (±6.7) vs. 29.7 (±6.2), *p* = 0.99 and women 31.4 (±6.2) vs. 31.5 (±6.8), *p* = 0.67). A higher proportion of women were overweight (37.6% vs. 29.0%), whereas obesity was more common among men (56.7% vs. 41.4%).

Unadjusted odds ratio for the sociodemographic variables found that men aged 45–60 years [0.7, 95% CI (0.5–0.9)] and those with household income of USD 2600–5000 [0.7, 95% CI (0.4–0.9)] were 30 times less likely to report unhealthy lifestyle. In women, unadjusted odds ratio found that those aged 45-60 years and 61-75 years were 1.23 (1.0, 1.5)] and 1.84 (1.4, 2.5) times respectively, more likely to report unhealthy lifestyle. Homemaker and those working in clerical jobs were 2.1 (1.1, 4.0) times more likely to report unhealthy lifestyle. 

Table 3 shows the frequency and significant difference between the barriers to physical activity and healthy diet in men and women. Generally, women reported fewer barriers than men; however, some barriers were equally reported by both men and women. Behavioral factors, like “lack of self-motivation” and “not enjoying physical activity,” were reported by around one-third of men and women. Similarly, family commitments were reported as a barrier for both physical activity and healthy eating by both genders. Moreover, certain environmental barriers like having no access to affordable places for physical activity were mentioned by both men and women.

Moreover, there was a significant difference in the frequency of barriers reported by men and women. Men most commonly reported that “harsh weather” (40.7%, *n* = 394) and “lack of time because of job commitments” (40.2%, *n* = 389) were barriers to physical activity, whereas “no access to healthy food” (40.1%, *n* = 388) and “not able to buy healthy food” (39.8%, *n* = 385) were most commonly reported as barriers to healthy eating. Women most frequently reported “no time because of family commitments” (36.3%, *n* = 737), followed by “no motivation to do physical activity” (33.2%, *n* = 674) and “don’t enjoy healthy food” (24.8%, *n* = 504), as barriers to physical activity and healthy eating, respectively. 

Table 4 shows the multivariate binary logistic model with adjusted odds ratio and 95% confidence interval (CI) for men and women. In men it was found that those aged 45–60 years [aOR 0.7, 95% CI (0.5–0.9)] and 61–75 years [aOR 0.6, 95% CI (0.4–1.0)] were at lower risk of reporting an unhealthy lifestyle, whereas men who do not enjoy physical activity [aOR 2.1, 95% CI (1.3, 3.4)], have no friend support to do physical activity [aOR 1.5, 95% CI (1.0 2.6)], or do not have enough information about a healthy diet [1.5, 95% CI (1.0, 2.0)] were at higher risk of reporting an unhealthy lifestyle. In women aged 45–60 [aOR 1.3, 95% CI (1.1, 1.7)] and 61–75 [aOR 2.4, 95% CI (1.7,2.3)], lack of motivation [aOR 1.3, 95% CI (1.1, 1.7)], feeling self-conscious while exercising [aOR 2.0, 95% CI (1.4, 2.9)], not enjoying healthy food [aOR 1.6, 95% CI (1.3, 2.1)], and no support from children in eating a healthy diet [aOR 1.4, 95% CI (1.1, 1.8)] were at higher risk of reporting an unhealthy lifestyle (Table 3). The model was adjusted for education level, occupation, and BMI for both men and women. 

## 5. Discussion

Unhealthy lifestyle may comprise of several factors, however, we focused on the behavioral factors, namely, physical activity, diet and smoking [22,23]. Looking at the results, it appears that almost half (47%) of the Saudi population is reporting an unhealthy lifestyle. The main contributing factors were low physical activity and unhealthy diet. Similar to previous studies, self-reported smoking prevalence was quite low in women, highlighting the fact that it may not be socially acceptable for women to smoke [24]. A significant population has already been diagnosed with a chronic disease or diseases and if combined with an unhealthy lifestyle, this may aggravate the existing health problems [25]. 

Overall, younger males and older aged women reported unhealthy lifestyle, and these differences suggest that no single strategy can be applied to the whole population [26]. The finding that older men were at less risk of an unhealthy lifestyle maybe because of successful efforts by healthcare workers [27]; however, it also suggests that health interventions with a focus on the young male population are required [28]. The opposite is required for women, because the number of women reporting an unhealthy lifestyle increased significantly after 60 years of age. This may be because of physiological changes in older women’s lives: for example, menopause, the onset of chronic diseases, or changes in family structure [29]. Hence, we suggest that age-specific strategies, for example work place based for the younger population and home based plan for the older population may encourage and support healthy lifestyle.

Besides age, certain behavioral, sociocultural and environmental factors were significantly associated with lifestyle. Different behavioral change models are available to address lack of motivation and lack of confidence [30]. However, we believe that an integrated model, inclusive of behavioral change, social support and environmental support, is required to deal with the factors associated with an unhealthy lifestyle. An integrated behavior change model can help with increasing self-motivation, social understanding, and reinforcing positive behavior [31,32]. Techniques such as regular feedback, the demonstration of a positive behavior or attitude, behavior practice or rehearsal, and grading the tasks can help with overcoming a lack of motivation and improve self-determination [31,32].

In addition, the uptake and maintenance of healthy lifestyle requires a supportive environment and policies to allow motivation to become an inherent part of one’s behavior [33]. Women participants reported that they feel self-conscious while exercising in front of others, which may be because of cultural restrictions or a lack of self-confidence [34]. Based on our findings we suggest that women require a specific allocated area and time to perform regular physical activity. This might instill confidence in women. According to the vision 2023, Saudi government is attempting to establish accessible exercise facilities in all major cities [12]. However, optimal and regular utilization of exercise facility is required to overcome these barriers. We found that, overall, 52% of the participants were obese; this is in close proximity to the future projected figures of 60% by 2022 [35]. Any behavior, to be sustainable, needs support from family, friends, and colleagues. The same stand for a healthy lifestyle: if there is positive support, physical activity and consuming a healthy diet can become enjoyable. However, negative peer pressure can be a distraction from regular physical activity and reason for unhealthy eating [36]. 

Women mostly report conflicting demands between work and childcare preventing them from adopting a healthy lifestyle [37]. The cultural norms expects that a Saudi woman will prioritize her family and household responsibilities over other engagements, such as going to a gymnasium for regular exercise [38]. While this may be culturally and socially approved, household chores will not always meet the recommended physical activity levels [39]. Hence, to overcome this barrier, an important initiative can be promoting PA at workplaces, which could be done by announcing health-promotion incentives (for example, free screening tests) or holding mandatory PA sessions on a regular basis [40]. Women mentioned that they do not have their children’s support in preparing healthy foods; we suggest that healthy cooking competitions based on innovative recipes should be arranged at school and community level to promote healthy cooking and eating habits [41,42]. Additional initiatives may include availability of social support groups for buying healthy groceries and availability of babysitters at places of exercise. Past studies have reported that avoiding physical activity because of extreme temperatures was one of the most common barriers [9,18,19]. However, we did not find a significant association between extreme weather and lifestyle, maybe because of the large number of indoor gymnasiums recently opened for both men and women [12]. 

More than one-third of the male participants lacked information about a healthy diet. It is commonly known that the consumption of fruit and vegetables is associated with several health benefits; however, consumption remains low in both developing and developed countries [43]. One of the reasons for the low consumption of fruit and vegetables could be a lack of attractive advertisements and information. Various social media channels can be utilized to disseminate information about healthy eating. Easy recipes based on fruit and vegetables should be advertised so that people are encouraged to prepare and consume a healthy diet instead of fast food and energy drinks [43]. 

Several campaigns across the world are promoting healthy food [44]. However, healthy lifestyle policies can only be successfully implemented if all stakeholders are involved in the decision-making [44]. Therefore, we suggest that all concerned ministries/institutes (health, education, social affairs. and recreation), join with the community to develop policies for promoting a healthy lifestyle. 

### Strengths and Limitations

The major strength of the current study is the large sample size, including various age groups, social classes and both working and nonworking population. We measured physical activity and dietary habits by utilizing a structured questionnaire. However, there were a few limitations. Although the PHCC were randomly selected, however the sample was self-selecting and we ended up with more number of women compared to men. It was a cross-sectional study, which prevents establishing any temporal relationship; thus, we cannot comment on the causality. We could not verify the smoking status in women, hence misreporting may have occurred. In addition, people belonging to rural areas may report different type of barriers, which we may have failed to capture. Hence results generalize to urban areas of Saudi Arabia. 

## 6. Conclusions

A significant percentage of the Saudi population has an unhealthy lifestyle. The factors associated with an unhealthy lifestyle differ according to age and gender. The current study offers several implications for clinical and public health practices in the Gulf region. Policymakers, health workers, and social scientists should develop policies and programs, keeping in consideration the differences between men and women, young adults and the older population. Specific strategies for women should focus on social support groups for the elderly, along with self-motivation, the allocation of protected time with easy access to exercise facilities and a separate space for exercise is required to promote physical activity for the younger population. The increasing number of working people highlights the need for environmentally friendly policies for initiating exercise programs at workplaces. 

## Figures and Tables

**Table 1:** The frequency of Saudi men and women reporting low levels of physical activity, inadequate dietary intake of vegetables and fruits, and smoking by age in Riyadh, Saudi Arabia.

	Age Group (in Years) ‡			Total (*n* = 2997) * Men = 968 ** Women = 2029	Odds Ratio
	30‒44	45‒59	60‒75		
Low Physical activity ^1^ Men Women Total	247 (56.9) 507 (50.1) 754 (52.2)	126 (29.0) 357 (35.3) 483 (33.4)	61 (14.1) 147 (14.5) 208 (14.4)	434 (44.8) 1011 (49.8) 1445 (48.2)	1.0 1.22 (1.05, 1.42)
<5 portions of vegetables and fruit/day ^2^ Men Women Total	137 (50.4) 235 (45.4) 372 (47.1)	84 (30.9) 215 (41.5) 299 (37.8)	51 (18.8) 68 (13.1) 119 (15.1)	272 (28.1) 518 (25.5) 790 (26.3)	1.0 0.93 (0.74, 1.17)
Regular Smoking ^3^ Men Women Total	209 (78.0) 31 (70.5) 240 (76.9)	46 (17.2) 10 (22.7) 56 (17.9)	13 (4.9) 3 (6.8) 16 (5.1)	268 (27.7) 44 (2.2) 312 (10.4)	1.0 0.03 (0.02, 0.04)

* Column percentage; ‡ Row percentage; ** Men were 1.49 (1.28, 1.74) times more likely to have an unhealthy lifestyle. ^1^ Low physical activity: <1200 METs min/week (*p* = 0.04). ^2^ <5 portions equal to <400 gms (*p* > 0.05); ^3^ *p* = 0.007.

**Table 2:** Bivariate analysis showing unadjusted odds ratio and 95% confidence interval for sociodemographic factors associated with an unhealthy lifestyle in Saudi men and women in Riyadh, Saudi Arabia.

Sociodemographic Characteristics	Men *N* = 968 (%)			Women *N* = 2029 (%)		
	Unhealthy Lifestyle *n* = 523(54%)	Healthy Lifestyle *n* = 445 (46%)	Unadjusted Odds Ratio 95% CI	Unhealthy Lifestyle 894 (44.1)	Healthy Lifestyle 1135 (55.9)	Unadjusted Odds Ratio 95% CI
**Age (in Years)** 30‒44 45‒60 61‒75	329 (62.9) 138 (26.4) 56 (10.7)	247 (55.5) 147 (33.0) 51 (11.5)	1.0 **0.7 (0.5, 0.9)** 0.8 (0.5, 1.2)	456 (51.0) 314 (35.1) 124 (13.9)	664 (58.5) 373 (32.9) 98 (8.6)	1.0 **1.23 (1.0,1.5)** **1.84 (1.4,2.5)**
**Marital Status** Single Married/divorced	58 (11.1) 465 (88.9)	44 (9.9) 401 (90.1)	1.0 0.9 (0.6,1.33)	69 (7.7) 825 (92.3)	77 (6.8) 1058 (93.2)	1.0 0.9 (0.6,1.21)
**Participant’s educational level** University or above High school or lower	377 (72.1) 146 (27.9)	337 (75.7) 108 (24.3)	1.0 1.2 (0.9, 1.61)	384 (43.0) 510 (57.0)	492 (43.3) 643 (56.7)	1.0 1.0 (0.8, 1.2)
**Educational level of spouse (men = 866, women = 1883)** University or above High school or lower	258 (55.5) 207 (44.5)	216 (53.9) 185 (46.1)	1.0 0.9 (0.7, 1.22)	349 (42.3) 476 (57.7)	412 (38.9) 646 (61.1)	1.0 0.8 (0.7, 1.04)
**Participant’s occupation** Professional Clerical Unemployed Retired Homemaker	287 (54.9) 175 (33.5) 14 (2.7) 47 (9.0) -	250 (56.2) 140 (31.4) 7 (1.6) 48 (10.8) -	1.0 1.1 (0.8, 1.4) 1.7 (0.7, 4,4) 0.8 (0.6, 1,3) -	14 (1.6) 343 (38.4) 35 (3.9) 26 (2.9) 476 (53.2)	46 (3.2) 425 (37.4) 68 (6.0) 33 (2.9) 573 (50.5)	1.0 **2.1 (1.1,3.9)** 1.3 (0.6, 2.8) 2.0 (0.9, 4.5) **2.1 (1.1, 4.0)**
**Spouse’s occupation** **(men = 866, women = 1883)** Professional Clerical Unemployed Retired Military service	362 (77.8) 79 (20.0) 6 (1.3) 18 (3.9) -	326 (81.3) 55 (13.7) 10 (2.5) 10 (2.5) -	1.0 1.3 (0.9, 1.9) 0.5 (0.2, 1.5) 1.6 (0.7, 3.5) -	175 (21.2) 301 (36.5) 37 (4.5) 203 (24.6) 109 (13.2)	226 (21.4) 317 (30.0) 40 (3.8) 310 (29.3) 165 (15.6)	1.0 **1.2 (1.0, 1.6)** 1.2 (0.7, 1.0S) 0.8 (0.6, 0.9) 0.9 (0.6, 1.2)
**Household Monthly Income (U.S. Dollars)** ≤2600 2600–5000 ≥5000	190 (36.3) 239 (45.7) 94 (18.0)	155 (34.8) 230 (51.7) 60 (13.5)	1.0 0.7 (0.4, 0.9) 0.8 (0.5, 1.1)	74 (8.3) 245 (27.4) 575 (64.3)	87 (7.7) 326 (28.7) 722 (63.6)	1.0 0.9 (0.6, 1.2) 0.9 (0.6, 1.3)
**Body Mass Index (kg/m^2^)** Normal (<25.0) Overweight (25.0‒29.9) Obese (≥30.0)	114 (21.8) 181 (34.6) 228 (43.6)	89 (20.0) 183 (41.1) 173 (38.9)	1.0 0.8 (0.5, 1.1) 1.0 (0.7, 1.4)	166 (14.8) 319 (28.5) 635 (56.7)	124 (13.6) 270 (29.7) 515 (56.7)	1.0 0.8 (0.6,1.2) 0.9 (0.7,1.2)

Significant odds ratio and 95% CI are given in bold.

**Table 3:** The frequency of Saudi men and women reporting barriers for low physical activity and unhealthy eating habits in Riyadh, Saudi Arabia.

Factors	Men *n* = 968 (%)	Women *n* = 2029 (%)	*p*-Value *
**Physical activity**			
No motivation to do physical activity	309 (31.9)	674 (33.2)	0.5
Don’t enjoy physical activity	260 (26.9)	520 (25.6)	0.5
Lack of appropriate skills to do physical activity	295 (30.5)	495 (24.4)	<0.001
No family support to do physical activity	309 (31.9)	466 (23.0)	<0.001
No friend/colleague support to do physical activity	355 (36.7)	363 (17.9)	<0.001
No family/ children support to do physical activity	306 (31.6)	380 (18.7)	<0.001
No time because of family commitments	368 (38.0)	737 (36.3)	0.3
No information on physical activity	299 (30.9)	343 (16.9)	<0.001
No access to places to do physical activity	299 (30.9)	653 (32.2)	0.5
No facilities that are affordable for physical activity	254 (26.2)	541 (26.7)	0.8
No time to do physical activity because of job	389 (40.2)	617 (30.4)	<0.001
Feel self-conscious/shy doing exercise in front of others	140 (14.5)	211 (10.4)	0.001
Not able to do physical activity because of weather	394 (40.7)	400 (19.7)	<0.001
Not able to do physical activity because of culture	275 (28.4)	340 (16.8)	<0.001
**Healthy eating**			
Not motivated to eat a healthy diet	313 (32.3)	450 (22.2)	<0.001
Don’t enjoy eating healthy food	335 (34.6)	504 (24.8)	<0.001
Don’t have the skills to prepare or cook healthy foods **	-	267 (13.2)	<0.001
No spouse/family support to eat a healthy diet	296 (30.6)	290 (14.3)	<0.001
No family/ children support to eat a healthy diet **	308 (31.8)	365 (18.0)	<0.001
No friend/colleague support to eat a healthy diet	377 (38.9)	274 (13.5)	<0.001
Not enough information about a healthy diet	366 (37.8)	318 (15.7)	<0.001
No access to healthy foods	388 (40.1)	262 (12.9)	<0.001
Not able to afford healthy foods	385 (39.8)	186 (9.2)	<0.001
No time to prepare or eat healthy foods because of job	-	384 (18.9)	0.03
No time to prepare or eat healthy foods because of family commitments	-	323 (15.9)	<0.001

*p value denotes whether there is a difference in frequency of reported barrier between men and women; **Questions asked of married women only.

**Table 4:** Multivariate logistic regression showing adjusted odds ratio and 95% CI for factors associated with an unhealthy lifestyle among Saudi men and women in Riyadh, Saudi Arabia.

Variables	Crude Odds Ratio (95% CI)	Adjusted Odds Ratio * (95% CI)	*p*-Value
**Men**			
Age (in years) 30‒44 45‒60 61‒75	1.0 0.2 (0.5, 0.9) 0.8 (0.5, 1.2)	1.0 0.7 (0.5, 0.9) 0.6 (0.4, 0.9)	0.03 0.05
Do not enjoy physical activity	2.3 (1.7, 3.1)	2.1 (1.3, 3.4)	0.003
No friend support to do physical activity	1.9 (1.5, 2.5)	1.5 (1.0, 2.6)	0.05
Do not have enough information about healthy diet	1.8 (1.4, 2.3)	1.5 (1.0, 2.0)	0.05
**Women**			
Age (in Years) 30‒44 45‒60 61‒75	1.0 1.2 (1.0, 1.5) 1.8 (1.4, 2.5)	1.0 1.3 (1.1, 1.7) 2.4 (1.7, 3.2)	0.004 <0.001
No motivation to do physical activity	1.3 (1.0, 1.5)	1.3 (1.1, 1.7)	0.008
Feel self-conscious/shy doing exercise in front of others	1.3 (1.0, 1.7)	2.0 (1.4, 2.9)	<0.001
Do not enjoy eating healthy food	1.6 (1.3, 1.9)	1.6 (1.3, 2.1)	<0.001
No family support to eat a healthy diet	1.4 (1.1, 1.7)	1.4 (1.1, 1.8)	0.01

* Multivariate models were adjusted for education level, occupation, and BMI.

## Data Availability

Data can be made available on special request to the principal Investigator.
